# Supplementary material for: A Simplified Treatment for Efficiently Modeling the Spectral Signal of Vibronic Transitions: Application to Aqueous Indole
Source: Molecules. 2022 Nov 22;27(23):8135. doi: 10.3390/molecules27238135 (PMC9739849; doi:10.3390/molecules27238135)
Supplement: Supplementary file 1 [file molecules-27-08135-s001.zip › molecules-2026860-supplementary.pdf]

Supplementary Materials:
A simplified treatment for efficiently modeling the spectral signal of vibronic transitions: application to aqueous Indole

Cheng Giuseppe Chen[1], Massimiliano Aschi[2], Marco D'Abramo[1,*], and Andrea Amadei[3,*]

S1 Benchmark of DFT functionals for the calculation of the gas phase vibronic transitions

The following functionals were considered to calculate the gas phase vibronic transitions of indole: B3LYP[1], CAM-B3LYP[2], PBE0[3], M06-2X[4] and ωB97X-D[5]. For each of those functionals we calculated the optimized geometries (both for the ground and the excited states) and their corresponding frequencies using TD-DFT and 6-311+G(d) as the basis set. All the calculations were performed using Gaussian16[6]. We show the results obtained for the geometry corresponding to the ground state minimum in Table S1.

Table S1: Gas phase excitation energies (ΔE) and electric transition dipole square length ($|\mu|^2$) for the ground state $\longrightarrow$ L_b and ground state $\longrightarrow$ L_a transitions of indole calculated using different functionals. We highlighted in bold the functionals in which the order of the L_b and L_a states is inverted compared to the results obtained with EOM-CCSD/6-311+G(d) (i.e. $\Delta E(L_b) < \Delta E(L_a)$, see main text).

	L_b		L_a	
	ΔE (eV)	$\|\mu\|^2$ (a.u.)	ΔE (eV)	$\|\mu\|^2$ (a.u.)
**B3LYP**	**4.8483**	0.2800	**4.7081**	0.6588
CAM-B3LYP	4.9920	0.3141	5.0388	0.8928
**PBE0**	**4.9476**	0.3154	**4.8229**	0.6784
M06-2X	5.0527	0.3951	5.1028	0.8294
ωB97X-D	5.0089	0.2906	5.0637	0.9129

From these results we immediately discarded B3LYP and PBE0 because of the inaccurate description of the L_b and L_a electronic states, in particular inverting the order of those states.

With the remaining functionals (i.e. CAM-B3LYP, M06-2X and ωB97X-D) we calculated the gas phase vibronic spectrum corresponding to the ground state $\longrightarrow$ L_b transition (see Figure S1) using the method implemented in Gaussian16[7,8] and no significant difference was observed. A larger basis set was also tested with M06-2X, namely aug-cc-pVTZ[9] (see Figure S1), which provided similar results.

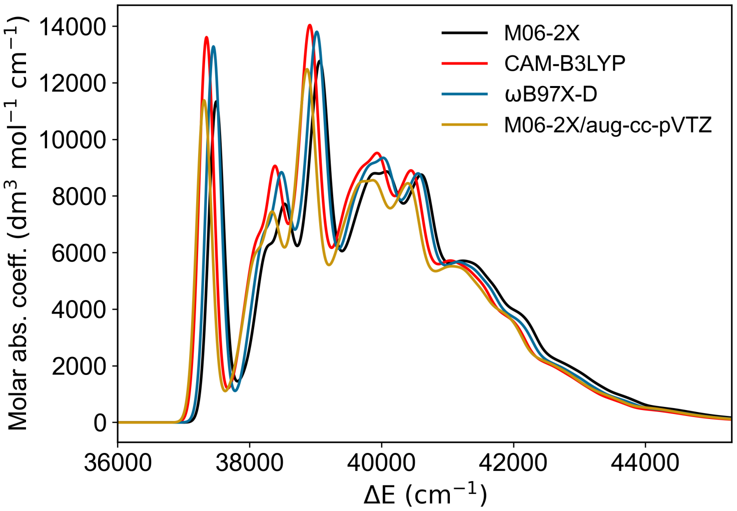

Figure S1: Gas phase vibronic spectra corresponding to the ground state $\longrightarrow L_b$ transition calculated using the method implemented in Gaussian16 (see main text) and M06-2X (black and yellow), CAM-B3LYP (red) and ωB97X-D (blue) as functionals and 6-311+G(d) as the basis set, expect when mentioned (in which case aug-cc-pVTZ was used).

References

[1] P. J. Stephens, F. J. Devlin, C. F. Chabalowski and M. J. Frisch, *J. Chem. Phys.*, 1994, **98**, 11623–11627.

[2] T. Yanai, D. P. Tew and N. C. Handy, *Chem. Phys. Lett.*, 2004, **393**, 51–57.

[3] C. Adamo and V. Barone, *J. Chem. Phys.*, 1999, **110**, 6158–6170.

[4] Y. Zhao and D. G. Truhlar, *Theor. Chem. Acc.*, 2008, **120**, 215–241.

[5] J.-D. Chai and M. Head-Gordon, *Phys. Chem. Chem. Phys.*, 2008, **10**, 6615–6620.

[6] M. J. Frisch, G. W. Trucks, H. B. Schlegel, G. E. Scuseria, M. A. Robb, J. R. Cheeseman, G. Scalmani, V. Barone, G. A. Petersson, H. Nakatsuji, X. Li, M. Caricato, A. V. Marenich, J. Bloino, B. G. Janesko, R. Gomperts, B. Mennucci, H. P. Hratchian, J. V. Ortiz, A. F. Izmaylov, J. L. Sonnenberg, D. Williams-Young, F. Ding, F. Lipparini, F. Egidi, J. Goings, B. Peng, A. Petrone, T. Henderson, D. Ranasinghe, V. G. Zakrzewski, J. Gao, N. Rega, G. Zheng, W. Liang, M. Hada, M. Ehara, K. Toyota, R. Fukuda, J. Hasegawa, M. Ishida, T. Nakajima, Y. Honda, O. Kitao, H. Nakai, T. Vreven, K. Throssell, J. A. Montgomery, Jr., J. E. Peralta, F. Ogliaro, M. J. Bearpark, J. J. Heyd, E. N. Brothers, K. N. Kudin, V. N. Staroverov, T. A. Keith, R. Kobayashi, J. Normand, K. Raghavachari, A. P. Rendell, J. C. Burant, S. S. Iyengar, J. Tomasi, M. Cossi, J. M. Millam, M. Klene, C. Adamo, R. Cammi, J. W. Ochterski, R. L. Martin, K. Morokuma, O. Farkas, J. B. Foresman and D. J. Fox, *Gaussian~16 Revision B.01*, 2016, Gaussian Inc. Wallingford CT.

[7] F. Santoro, A. Lami, R. Improta, J. Bloino and V. Barone, *J. Chem. Phys.*, 2008, **128**, 224311.

[8] V. Barone, J. Bloino, M. Biczysko and F. Santoro, *J. Chem. Theory Comput.*, 2009, **5**, 540–554.

[9] R. A. Kendall, T. H. Dunning and R. J. Harrison, *J. Chem. Phys.*, 1992, **96**, 6796–6806.
